# Comparative genomic analyses illuminate the distinct evolution of megabats within Chiroptera

**DOI:** 10.1093/dnares/dsaa021

**Published:** 2020-09-23

**Authors:** Masato Nikaido, Shinji Kondo, Zicong Zhang, Jiaqi Wu, Hidenori Nishihara, Yoshihito Niimura, Shunta Suzuki, Kazushige Touhara, Yutaka Suzuki, Hideki Noguchi, Yohei Minakuchi, Atsushi Toyoda, Asao Fujiyama, Sumio Sugano, Misako Yoneda, Chieko Kai

**Affiliations:** 1 School of Life Science and Technology, Tokyo Institute of Technology, Meguro-ku, Tokyo 152-8550, Japan; 2 Advanced Genomics Center, National Institute of Genetics, Mishima, Shizuoka 411-8540, Japan; 3 Joint Support-Center for Data Science Research, Research Organization of Information and Systems, Mishima, Shizuoka 411-8540, Japan; 4 Department of Computational Intelligence and Systems Science, Tokyo Institute of Technology, Yokohama, Kanagawa 226-8502, Japan; 5 School of Life Science and Technology, Tokyo Institute of Technology, 4259 Nagatsuta-cho, Midori-ku, Yokohama, Kanagawa 226-8501, Japan; 6 Department of Applied Biological Chemistry, Graduate School of Agricultural and Life Sciences, The University of Tokyo, Tokyo 113-8657, Japan; 7 Department of Medical Genome Sciences, Graduate School of Frontier Sciences, The University of Tokyo, Kashiwa City, Chiba 277-0882, Japan; 8 Comparative Genomics Laboratory, National Institute of Genetics, Mishima, Shizuoka 411-8540, Japan; 9 Institute of Medical Science, The University of Tokyo, Minato-ku, Tokyo 108-8639, Japan; 10 Institute of Industrial Science, The University of Tokyo, Meguro-ku, Tokyo 153-8505, Japan

**Keywords:** megabat, whole genome, adaptive evolution, SINEs, chemosensory receptor genes

## Abstract

The revision of the sub-order Microchiroptera is one of the most intriguing outcomes in recent mammalian molecular phylogeny. The unexpected sister–taxon relationship between rhinolophoid microbats and megabats, with the exclusion of other microbats, suggests that megabats arose in a relatively short period of time from a microbat-like ancestor. In order to understand the genetic mechanism underlying adaptive evolution in megabats, we determined the whole-genome sequences of two rousette megabats, Leschenault’s rousette (*Rousettus leschenaultia*) and the Egyptian fruit bat (*R. aegyptiacus*). The sequences were compared with those of 22 other mammals, including nine bats, available in the database. We identified that megabat genomes are distinct in that they have extremely low activity of SINE retrotranspositions, expansion of two chemosensory gene families, including the trace amine receptor (*TAAR*) and olfactory receptor (*OR*), and elevation of the *dN*/*dS* ratio in genes for immunity and protein catabolism. The adaptive signatures discovered in the genomes of megabats may provide crucial insight into their distinct evolution, including key processes such as virus resistance, loss of echolocation, and frugivorous feeding.

## 1. Introduction

Bats belong to the order Chiroptera and have the ability of powered flight. Accounting for one-fifth of all mammals in terms of the number of species, bats are one of the most successful groups of mammals.^1^ It is of primary interest for biologists to identify the processes and mechanisms of dynamic adaptation in bats. Traditionally, morphological and paleontological analyses placed the order Chiroptera within the superorder Archonta (Primates, Dermoptera, Chiroptera, and Scandentia).^2^ However, DNA sequencing data has challenged the validity of the Archonta, and alternatively proposed the inclusion of bats into Laurasiatheria (Cetartiodactyla, Perissodactyla, Carnivora, Pholidota, Chiroptera and Eulipotyphla).^3–6^ Although Laurasiatheria is now considered to be a natural assemblage, the phylogenetic position of bats within Laurasiatheria remains to be resolved.^7^^,^^8^

The paraphyly of microbats is also under debate. Traditionally, morphological studies proposed the sub-division of the order Chiroptera into two suborders: Microchiroptera (microbats) and Megachiroptera (megabats or Old-World fruit bats).^9^ Microbats use ultrasonic echolocation for flight and for foraging in the night, whereas megabats do not echolocate, and primarily use vision to fly and feed on fruits and/or nectars. Megabats are also neuro-anatomically distinct from microbats, as megabats have a developed visual system.^10^ Molecular data suggests that five lineages of microbats, including Rhinopomatidae, Rhinolophidae, Hipposideridae, Craseonycteridae, and Megadermatidae, are more closely related to megabats than to other microbats. Therefore, the five lineages of rhinolophoid microbats and megabats were re-classified as ‘Yinpterochiroptera’ and the remaining microbats as ‘Yangochiroptera’.^5^^,^^11^^,^^12^ Thus, recent molecular studies suggest that several adaptive characteristics specific to megabats have emerged within a short period of time from a microbat-like ancestor.

Genome-wide analyses have been used to identify the unique evolution of bats in several studies. Seim *et al*.’s^13^ study determined the genome sequence of one microbat (Brandt’s bat) and found the signatures for adaptive evolution in genes related to physiology and longevity. Zhang *et al*.^14^ determined the genome sequences of one microbat (David’s myotis) and one megabat (black flying fox) and found that genes for flight and immunity evolved due to positive selection. Parker *et al*.^15^ identified the genomes of three microbats, including the greater horseshoe bat, the greater false vampire bat, and Parnell’s mustached bat, and one megabat, the straw-coloured fruit bat. In comparing the genomes of these bats with those of other mammals, this study identified that genes related to hearing/deafness showed convergent evolution among echolocating mammals. Pavlovich *et al*.^16^ recently determined the whole genome of the Egyptian fruit bat (*R. aegyptiacus*), which is a natural reservoir for the Marburg virus, and revealed that the genes for immunity were expanded and diversified, suggesting an antiviral mechanism that is used to control viral infection. Especially, as bats are natural hosts for zoonotic virus including henipaviruses, filoviruses, and coronaviruses, which are emerging viruses with high rates of fatality, the comparative genomic study in bats may provide an effective solution against the current global pandemics of coronavirus disease-2019 (COVID-19).^17^

In this study, we determined the genome sequences of two rousette megabats, Leschenault’s rousette (*Rousettus leschenaultia*) and the Egyptian fruit bat (*R. aegyptiacus*). We assessed the genomic signatures for the process of natural selection that facilitates the dynamic and adaptive evolution of megabats. In particular, the main aim to determine the whole-genome sequence of Egyptian fruit bat in addition to the previous study^16^ is to obtain higher quality genome data, which facilitates more accurate and comprehensive gene annotations, especially for multi-gene families. In addition, the genome sequences of Leschenault’s rousette belonging to the same genus as the Egyptian fruit bat is of our interest to identify genomic differences in closely related bat species. These genome sequences were compared with those of 22 mammals, including six microbats and three megabats, available in the database. We used genome-wide phylogenetic analyses, followed by candidate gene analyses focussed on retroposons and chemosensory multi-gene families for taste, olfaction, and pheromone detection. In addition, we also performed global positive selection analyses. As a result, the inter-relationships among Laurasiatheria were consistently reconstructed, with the order Eulipotyphla diverging first, followed by the divergence of Chiroptera and the remaining groups, including Cetartiodactyla, Perissodactyla, Pholidota, and Carnivora. The reciprocal monophyly of Yinpterochiroptera and Yangochiroptera was also shown with reliable statistical support. We revealed several notable distinct features in megabat genomes, including extremely low activity of SINE retrotranspositions and the expansion of the genes for the trace amine receptor (*TAAR*) and olfactory receptor (*OR*). Additionally, the signatures for positive or relaxed selection were observed in genes for immunity and protein catabolism. Thus, our comparative genomic analyses may illuminate the genetic mechanisms underlying the dynamic adaptation of megabats during diversification in the order Chiroptera.

## 2. Materials and methods

### 2.1. Specimens and tissue samples

Egyptian fruit bats (*R. aegyptiacus*) and Leschenault’s rousettes (*R. leschenaulti*), both of which were provided by Ueno Zoo, were maintained under controlled conditions using an air conditioner and moisture chamber. The animals were kept in steel cages and fed fruit and water at the same time every day. All experiments were performed in accordance with the Animal Experimentation Guidelines of the University of Tokyo and were approved by the Institutional Animal Care and Use Committee of the University of Tokyo. As for Egyptian fruit bats, we prepared kidney-derived primary cultured cells. A pregnant Egyptian fruit bat was deeply anesthetized with isoflurane, the uteri were surgically removed, and the animal was euthanized by bleeding. The kidney from the fetus was fragmented using scissors and treatment with TrypLE (Gibco). The fragmented kidney was then cultured in DMED containing 5% fetal calf serum to obtain primary cultured cells.

### 2.2. Genome sequencing and assembly

Genomic DNA was extracted from the frozen spleen tissue or cultured kidney cells of two individuals of Egyptian fruit bat, and frozen kidney tissue from one individual of Leschenault’s rousette, using a Blood & Cell Culture DNA Kit (Qiagen, Hilden, Germany), according to the manufacturer’s protocol with minor laboratory customizations, the information can be available upon request. The DNA samples (>20 kb) were subjected to the sequencing as described below after quality and quantity check. To construct paired-end sequencing libraries, the genomic DNA was fragmented using a Covaris S2 Focussed-ultrasonicator (Covaris, Woburn, MA, USA). The paired-end libraries were constructed using the TruSeq DNA PCR-Free Library Prep kit (Illumina, San Diego, CA, USA). Mate pair libraries were prepared from genomic DNA using the Nextera Mate Pair Sample Preparation Kit (Illumina, San Diego, CA, USA). All libraries were sequenced on an Illumina-HiSeq 2500 system using rapid-mode chemistry with paired-end sequencing. Prior to assembly, data pre-processing was performed. First, the adapter sequences were trimmed using the fastq-clipper ea-utils v1.1.2,^18^ setting the parameters to ‘-p 10 -m 1 -l 0’. Second, we filtered the reads mapped to the mitochondrial genome using BWA-ALN v0.6.2^19^ with default parameters. Finally, we performed base error correction using SOAPec v2.01^20^ with the parameters ‘-k27 -L 150’. We then assembled the reads using Platanus v1.2.1^21^ with default parameters. Contamination candidates were removed by mapping to *Escherichia coli* and *PhiX* genomes using blastn v2.2.9,^22^ setting the parameters to ‘-e 1e-30’. The statistics of the genome assemblies and the information of sequence libraries are summarized in Supplementary Tables S1-1 and S1-2. In order to test the quality of the reference assembly in the Egyptian fruit bat, we additionally constructed a fosmid library, which was end-sequenced using ABI 3730xl sequencers.

### 2.3. Identification of protein-coding genes in bat genomes

The protein-coding genes in the genomes of Egyptian fruit bat and Leschenault’s rousette were identified based on the alignment with annotated gene sequences of 14 mammals (cat, dog, horse, cow, hedgehog, human, macaque, mouse, rat, Black flying fox, Little brown bat, Brandt’s bat, David’s myotis, and Large flying fox; Supplementary Table S2) that are available in the database. The sequences for each gene of the 14 mammals were aligned to the two bat genomes by using BLAT^23^ to identify approximate gene loci. The BLAT alignments of the gene sequences to the genomes were refined by the exonerate software to estimate the exon–intron boundaries.^24^

In addition to the homology-based identification, RNA-seq-based transcript reconstruction and *ab initio* gene prediction were performed to identify the protein coding genes. RNA of primary culture cells from the kidney of the Egyptian fruit bat was extracted by using TRIzol reagent (Thermo Fisher). A total of 122,017,734 paired-end reads of mRNA (Illumina-HiSeq, 101 bp) were aligned to the genomes using tophat.^25^ In total, 97,696,475 and 88,528,280 paired-end reads could be mapped to the genome sequences of *R. aegyptiacus* and *R. leschenaultii*, respectively. Transcript structures were reconstructed using AUGUSTUS^26^ based on the tophat alignment of the Illumina reads to the bat genomes. The expression levels of the reconstructed genes were computed using cufflinks^27^ based on the tophat alignment of the Illumina reads to the genomes. A total of 8,079 genes were expressed with fragments per kilobase of transcript per million of reads mapped (FPKM) ≥ 1 in the kidney-derived primary cultured cells. Examples are shown in Supplementary Fig. S1. *Ab initio* genes were obtained by using Genscan^28^ and SNAP.^29^ The genomic sequences were cut to seven megabase-long fragments, and Genscan was run on each fragment. The genes identified were assigned to gene loci based on the overlap of exons on the same strand, and the redundancies of the transcripts were removed. Only transcripts annotated with the start codon (ATG) and introns flanked by canonical splice dinucleotide pairs (GT-AG, GC-AG, and AT-AC) were retained. A total of 46,249 and 47,073 transcripts were annotated over 20,005 and 20,913 gene loci, respectively, on the genomes of the Egyptian fruit bat and Leschenault’s rousette. The completeness of the gene determination was evaluated by using BUSCO.^30^ Similarly, we assessed protein-coding genes on the genomes of four other bat species, the Straw-coloured fruit bat, the Greater false vampire bat, the Greater horseshoe bat, and the Straw-coloured fruit bat.^15^ Due to fragmental nature of these genome assemblies (N50: 15–27 kb), however, we did not use the thresholds of initial codon and splice sites as used in the annotation of the genomes of the Egyptian fruit bat and Leschenault’s rousette. We identified the longest ORF in each transcript mapped by exonerate by using TransDecoder (https://github.com/TransDecoder/TransDecoder/blob/master/TransDecoder.LongOrfs) and used it as the gene annotation. We identified 28,367–31,441 transcripts in 19,296–20,272 gene loci on these genomes (Supplementary Table S3). The ratio of complete genes of the annotated genes evaluated by BUSCO was 53.8–76.0%.

Additional to this annotation, the tandemly duplicated receptor genes, including *OR*s, taste receptors (*T1R*s and *T2R*s), vomeronasal receptors (*V1R*s and *V2R*s), formyl peptide receptors (*FPR*s), and *TAAR*s, were annotated separately. Olfactory receptors were identified by the method described previously.^31^^,^^32^ The other receptor genes were identified using another protocol.^33^ In short, we obtained protein sequences of mammalian *T1R*s, *T2R*s, *FPR*s, *V1R*s, *V2R*s, and *TAAR*s from the NCBI RefSeq database (https://www.ncbi.nlm.nih.gov/refseq/). The redundant sequences, which contain more than 80% identity as identified by CD-HIT^34^, were removed to establish representative query sequences. For *T1R*s, we used only the transmembrane regions as query sequences. We used the NCBI Conserved Domains database to annotate the 7-transmembrane domains of *T1R*s. Using the query sequences, we performed a tblastn search against the whole-genome sequence assemblies available in GenBank (https://www.ncbi.nlm.nih.gov/genbank/). The taxonomic classification and the accession numbers of the whole-genome sequences are summarized in Supplementary Table S2. The exon–intron structure of each sequence, which was obtained by tblastn, was predicted with the exonerate program^24^ using translated query sequences as protein models. The resulting hit sequences were classified into ‘intact’, ‘truncated’, and ‘pseudo-genes’. Due to an assembly issue, the ‘truncated’ genes included poly ‘N’ sequences. In order to estimate the gene copy numbers, in these analyses, we treated the ‘truncated’ genes as ‘putatively intact’. The pseudo-genes include inactivating mutations in the coding region. The resulting genes were assessed to determine whether they encode the chemosensory receptors of interest using blastx searches and GHOSTZ^35^ against the UniRef^50^ database (https://www.uniprot.org/help/uniref). We used the Framework for Annotating Translatable Exons (FATE), which is available in GitHub (https://github.com/Hikoyu/FATE), for the automation of the procedures described above.

### 2.4. Phylogenetic tree construction

We constructed a phylogenetic tree based on the single-copy orthologous gene sets of mammals, as previously reported by Wu *et al*.,^36^ to elucidate the phylogenetic relationships of megabats with other mammals. Briefly, the nucleotide sequences of the 6,365 protein-coding genes of the two megabat species and 22 other mammalian species (Supplementary Table S2) were aligned using the PRANK software v.170427^37^ in codon level. Sites that are shared by <70% of the species were removed from the alignment. Among the 6,365 genes, 2,093 genes were listed for all species, and were used for the analyses. The gene tree was constructed using RAxML software, v8.1.12^38^ using the GTR+Γ+I model with 1,000 bootstrap replicates for each 2,093 gene. We collected the best tree for all 2,093 genes, which were used to infer the coalescent species tree with branch length by ASTRAL-III.^39^ The node support of the species tree was obtained by 1,000 replicates of bootstrapping. Branch length shown in the tree indicates the branch length in coalescent units.^40^

### 2.5. Repeat analysis

We used the genome of Leschenault’s rousette for the identification of TEs, based on two approaches, including *de novo* characterization of TEs and identification of homologous copies of known TEs in another megabat, the large flying fox (Supplementary Table S2). In the first approach, RepeatModeler ver. 1.0.8 (http://www.repeatmasker.org/RepeatModeler.html) was used to obtain a collection of repetitive sequences. For each of the preliminary consensus sequences, we conducted a local nucleotide blast search (*r* = 2, *G* = 5, *E* = 2, with an *e*-value cutoff of 10^−10^) and collected 80–100 copies along with their 10-kp flanking sequences. The copy sequences were aligned using MAFFT ver. 7.^41^ The alignment was manually modified using MEGA 5.0^42^ and a consensus sequence was re-constructed. The consensus sequence was used for the next round of the blast search, as described above, to obtain additional copies. This procedure was repeated until a full-length consensus sequence was completed. The full-length TEs were characterized and classified based on the sequence structure, including terminal inverted repeats and long terminal repeats (LTRs), coding proteins such as transposase and reverse transcriptase, and by comparison with known elements using RepeatMasker ver. 4.0.6 (http://repeatmasker.org), CENSOR,^43^ and RTclass1.^44^ For the second approach, a TE library of another megabat, the large flying fox, including 65 TE families which were obtained from RepBase,^45^ was used as a query for a homology search against Leschenault’s rousette. The local nucleotide blast search, alignment of the copy sequences, and reconstruction of the consensus sequence were conducted as described above. A similar blast search was also conducted using 102 TE families of a microbat (the little brown bat, Supplementary Table S2) library; however, no additional novel TEs were found except in the results from the two approaches listed above. All of the newly characterized 118 TE (sub) families were designated in conformity with the RepBase classification.

The repeat contents of the two *Rousettus* genomes were estimated using RepeatMasker with the sensitive option (-s) of cross-match search using the *Rousettus* repeat library which we developed here. The TE contents, such as the number of copies and length, were summarized based on their divergence (%) from the consensus sequence at the family/subfamily levels by using in-house Perl scripts. The TE contents of other species were summarized based on the RepeatMasker output (http://www.repeatmasker.org/genomicDatasets/RMGenomicDatasets.html).

### 2.6. Detection of positively selected genes in the fruit bats

**Figure 1:**
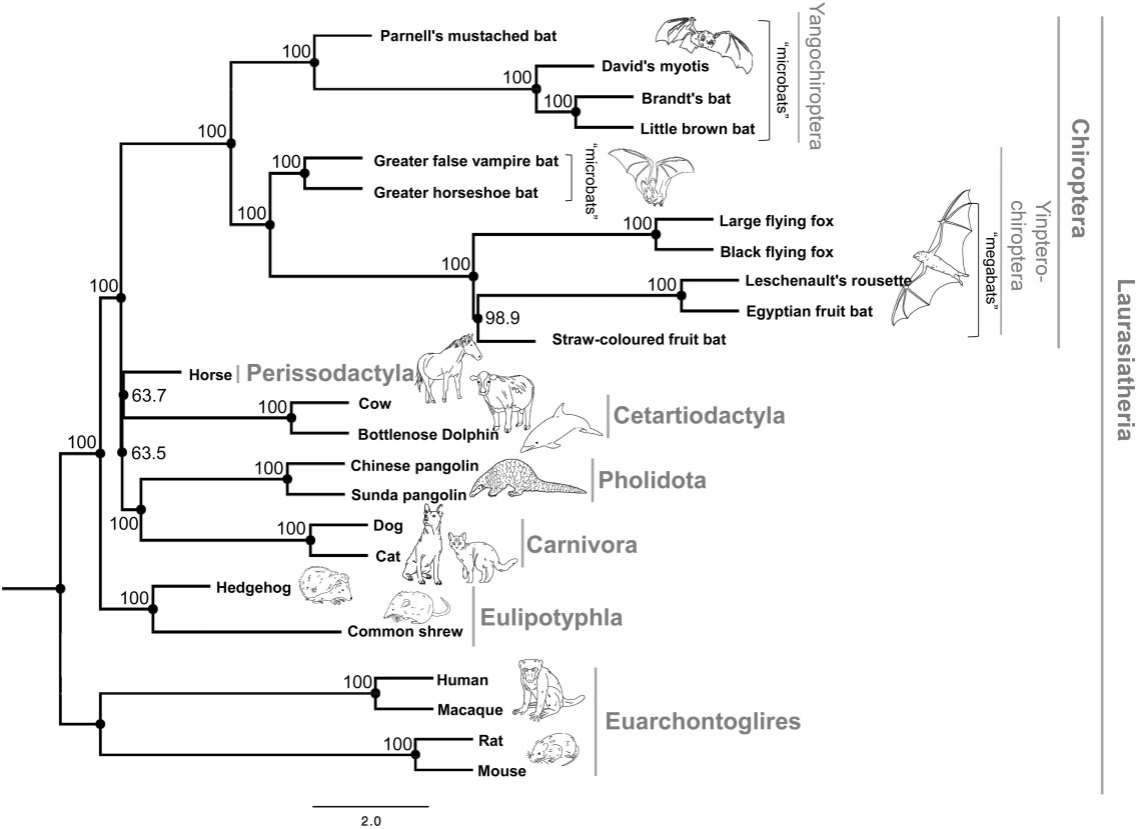
Genome-wide phylogenetic tree of 24 mammals, including 11 species of bats. Maximum likelihood tree of 24 mammals with branch length, based on the 2,093 single-copy orthologous gene set. The numbers in each node indicate the BP obtained by 1,000 times sampling of bootstrapping. The classification of the superorder, order, and sub-order is shown by the gray vertical bars. The scale bar indicates branch length in coalescent units.^40^

Orthologous genes under relaxed selection on megabat lineages were identified from the aligned 6,365 single-copy genes. On every alignment, we used the codeml branch model in PAML 4.8^46^ to detect the elevation of the *dN*/*dS* ratio (the non-synonymous substitution rate to the synonymous substitution rate) on stem and crown megabat branches. The species tree shown in Fig. 1 was used as a guide tree in the analysis. Likelihood ratio tests and inspections of the *P*-value were used to compare likelihoods between two models: (i) that assumed the megabat lineages as foreground branches; and (ii) that assumed the *dN*/*dS* ratio was not altered in all branches (null hypothesis), to evaluate the significance of the elevation of the *dN*/*dS* ratio for megabat branches. We performed further analyses for the genes of interest using the codeml branch-site models for analysing the positive selection on each site. In the branch-site test, we tested stem and crown megabats as the foreground branches and used microbats and outgroup species, including human, macaque, mouse, rat, cat, dog, Chinese pangolin, Sunda pangolin, bottlenose dolphin, cow, horse, hedgehog, Asian musk shrew, and common shrew, as background branches. For the branch-site test, we used two models for the analysis, including one model of a null hypothesis that assumes that the gene was under two types of selective pressures (purifying selection and neutral selection), and one model that used an alternative hypothesis to assume the gene was under three categories of selective pressures, including positive selection on the megabat branches. The likelihood ratio test comparing the likelihoods of these two models was used to evaluate the significance of the alternative model. To assess the functionality of positively selected sites, protein structure deposited in protein data bank (PDB) was used. The protein structures were depicted using the open-source version of PyMOL.^47^

## 3. Results and discussion

### 3.1. Genome assembly

We constructed draft genomes of the Egyptian fruit bat and Leschenault’s rousette by assembling short read data into contigs and scaffolding them using Platanus v1.2.1.^21^ The genome of the Egyptian fruit bat is composed of 1.90 Gbp with 4,974 scaffolds (*N*_50_ = 37.2 Mbp) and the genome of Leschenault’s rousette is composed of 1.90 Gbp with 8,141 scaffolds (*N*_50_ = 32.7 Mbp) (Supplementary Tables S1-1 and S1-2). The high qualities of the two genomes are demonstrated by the ratios of complete genes, which are 98.1 and 97.9%, respectively, as evaluated by BUSCO^30^ (Supplementary Table S3). The quality of both genomes in terms of the continuity of the scaffolds and the rate of *N* is high enough to facilitate genome-wide evolutionary analyses and characterization of multi-gene families. In addition, independent genome assemblies and gene annotations of the two individuals of Egyptian fruit bat determined in the previous study^16^ and this study may be utilized as an initial step towards the identification of the genotypic, transcriptomic, and phenotypic variation of this species in the future research.

### 3.2. Phylogenetic relationships of bats among mammals

Figure 1 shows the maximum likelihood phylogenetic tree with the time scale for 24 mammals, including 11 bats (five megabats and six microbats) based on 2,093 single-copy orthologous gene sets. Four species of Euarchontoglires, including humans, macaca, mouse, and rat, were used as outgroups. The tree successfully highlights the evolutionary history of Laurasiatherian mammals in that Eulipotyphla diverged first among them. In this phylogenetic tree, Chiroptera diverged after Eulipotyphla; however, the bootstrap probability (BP) supporting this node was not so high (63.5). In addition, the grouping of Pegasoferae (Chiroptera, Perissodactyla, and Carnivora), which was originally proposed by the insertion of retroposons^7^ and supported by several genome-wide analyses,^13^^,^^14^ was not supported. Given that the BPs for the inter-relationships of Cetartiodactyla, Perissodactyla (Carnivora + Pholidota), and Chiroptera were relatively low (63.7, 63.5) and the branch lengths were markedly short, it is highly likely that the initial divergence of Laurasiatherian mammals occurred rapidly during evolution. Such rapid speciation events may hamper reconstruction of the consistent tree topology for these groups.^8^^,^^48^ Importantly, as it was shown in the previous studies,^5^^,^^49^^,^^50^ the reciprocal monophyly of Yangochiroptera and Yinpterochiroptera was successfully supported in this analysis, suggesting that the megabats are nested in microbat lineages. Although it is difficult to estimate the ancestral state in the megabat ancestors due to the rarity of the fossil record, the phylogenetic tree suggests that several distinct characteristics in megabats, including the well-developed visual system, frugivorous diet, and the absence of echolocation, evolved in a short period of time during evolution from a ‘microbat-like’ ancestor. We next focussed on assessing the signatures for such adaptive evolution in these groups based on the genome-wide comparative analyses.

### 3.3. TEs in the two *Rousettus* genomes

**Figure 2:**
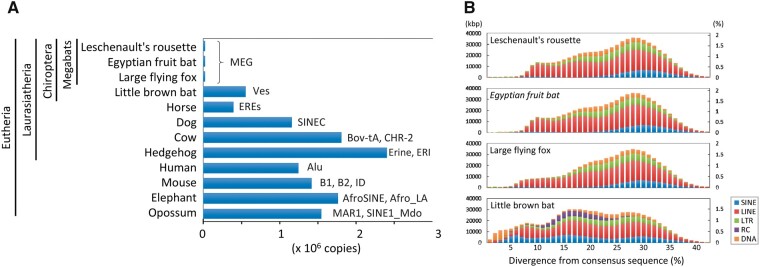
Low copy number of recently (retro-)transposed SINEs in megabats. (A) Copy number of clade-specific SINEs (i.e. excluding common SINEs, such as MIRs, AmnSINEs, and LF-SINEs) were compared among the three megabats, one microbat, and eight other mammals. Representative SINE families in each mammalian clade are shown. (B) Age distribution of occupied length (left) and proportion (right) of TE classes (SINE, LINE, LTR retrotransposons, rolling-circle transposons [RC], and DNA transposons) in the four bat genomes. Copies of lower divergence from the consensus sequence represent TEs inserted more recently.

In both the Leschenault’s rousette and Egyptian fruit bat genomes, TEs account for ∼35% of the genome, including SINEs (3.9%), LINEs (21%), LTR retrotransposons (6.2%), and DNA transposons (4.0%) (Supplementary Table S4 and Fig. S2). It is notable that the proportions of TEs in megabats, including the two *Rousettus* species and the large flying fox, are considerably lower as compared to the levels in other mammals, such as humans, where nearly half of the genome is covered by TEs (Fig. 2A and Supplementary Fig. S2). Consistent with the previous observations, it is also interesting that the proportion of TEs is generally correlated with the genome size in mammals^51^^,^^52^ (Supplementary Fig. S2). Co-variation between an accumulation of TEs and DNA loss by large segmental deletions is considered a major contributing factor to determine the genome size.^50^ Therefore, the smaller genome sizes in the megabats may be due to a lower activity of TEs, at least in part. Indeed, our analysis revealed that the number of young (recently retrotransposed) TE copies in the megabat genomes is very small (Fig. 2B and Supplementary Fig. S3). As exemplified by the microbat *Myotis lucifugus*, where the number of TEs representing <5% divergence from the consensus sequence is 180,000 (6.5% among all TE copies; Supplementary Fig. S3) consistent with the previous studies,^53^ in general, young TEs constitute a few percent among all TEs in mammalian genomes. However, the copy numbers of young TEs is only 2,900 (0.15%) and 7,300 (0.38%) for the *Rousettus* species and large flying fox, respectively (Fig. 2BSupplementary Fig. S3).

The small proportion of young TEs is partly accounted for the low frequency of retrotransposition events in megabat-specific SINEs (Fig. 2). In general, different types of SINE families are distributed for each mammalian clade, such as order, sub-order, or family.^52^ In megabats, the only known active SINEs are the 5S rRNA-derived MEG SINEs.^54^ It should be noted that *Rousettus* genomes contain no more than 23,000 copies of the MEG-related SINEs, which cover 0.21% of the genome. However, clade-specific SINEs are, in general, retrotranspositionally highly active, with 10^5^–10^6^ copies present in each mammalian genome (Fig. 2A). The large flying fox (*Pteropus vampyrus*) also has only 22,000 copies of MEG-related SINEs. Based on the wide distribution of MEG SINEs in megabats, including *Rousettus*, *Macroglossus*, *Eonycteris*, and *Cynopterus*,^54^ the origin of MEG can be traced back to the common ancestor of megabats, which existed at least 24 million years ago.^49^ It is possible that such a low retrotranspositional activity of the SINEs found in *Rousettus* and *Pteropus* is observed widely among megabats. It has been demonstrated that flying vertebrates, including bats, have substantially lost TEs and have smaller genome sizes in association with cellular metabolic constraints.^55^^,^^56^ The small proportion of MEG SINEs in the megabats may also be a result of the constraint related to their powered flight.

Another notable TE family is LINE-1 (L1), as it has been reported that the retrotranspositional activity of L1 has been lost in megabats.^57^ It is unlikely that the extinction of L1 resulted from the quiescence of L1 itself, because a synthesized sequence of the reconstructed megabat L1 is capable of retrotransposition in human HeLa cells.^58^ In addition, we identified that in addition to L1, all types of TEs have the least activity in megabats among the mammals investigated (Fig. 2B). This low activity of young TEs may be due to an unknown megabat-specific mechanism for TE repression or a result of extensive DNA loss during the past tens of millions of years.

One of the possible mechanisms by which TE activity may be tightly repressed is an antiviral immune system in megabats. Suggesting that the Egyptian fruit bat may possess a novel mode of antiviral defense,^16^ several antiviral-related genes are known to have expanded in this bat. For example, ribonuclease L, an interferon-inducible endoribonuclease that cleaves viral RNAs,^59^ evolved under relaxed selective constraint in bats.^16^ Ribonuclease L is also known to restrict retrotransposition of human L1 and mouse IAP elements in human cells.^60^ In addition, several other factors that restrict retrotransposition in humans and mice are known to be involved in an antiviral immune system.^61^ Thus, it is possible that a unique antiviral mechanism against exogenous parasites (i.e. viruses) is secondarily used for the restriction of the endogenous retroelements. As general mobilization of SINEs in mammals relies on the L1 machinery, the restriction of megabat L1 could limit the MEG SINE activity.^62^ The low activity of TEs may partly contribute to the small genome size (Supplementary Fig. S2), which could also be advantageous with respect to cell size and metabolic constraints in megabats as well as other flying vertebrates^55^^,^^56^. Therefore, the unusual characteristics of the TEs, likely shared among megabats, are an important example to study the molecular mechanisms underlying restriction of retrotransposition. Such future studies may shed light on the reason why bats have such compact genomes. It also remains unknown why Ves SINEs in microbats are active, whereas the genome size is relatively small among mammals (Fig. 2). The difference in the SINE activity between megabats and microbats may be affected by a possibly distinct antiviral immune system between the two groups, given that expansion of some antiviral-related genes occurred specifically in megabats.^16^

### 3.4. Chemosensory receptor genes (taste, olfaction, and pheromone)

Most of the chemosensory receptors are encoded by multi-gene families, allowing animals to detect highly diversified chemicals in the environment. The previously published studies have shown that the collections of the chemosensory receptor genes are flexible and highly variable among mammals, including the ORs, taste receptors (*T1R*s and *T2R*s), vomeronasal receptors (*V1R*s and *V2R*s), *FPR*s, and *TAAR*s.^63^ The number of certain chemosensory receptor gene families has been shown to have a strong correlation with the degree of dependence on these ligand chemicals for survival.^32^^,^^64^^,^^65^ Several studies have revealed that bats lost several chemosensory receptor genes, such as *T1R1* for umami,^66^ and *V1R*s for pheromone(s)^67^ that may be due to the specific sensory adaptation in the ancestor of these groups. It is possible that megabats re-allocated the diversity in chemosensory receptor genes as a sensory trade-off, given that megabats have experienced the secondary loss of echolocation ability, which is one of the most specialized senses in bats.^68^ To examine this possibility, we comprehensively characterized the chemosensory receptor genes and compared their diversity by focussing on whether or not the repertoires in megabats show notable differences from those in microbats.

**Figure 3:**
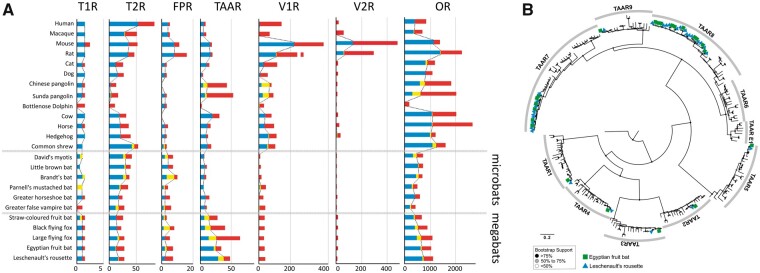
Comparison of the copy numbers of seven chemosensory receptor genes for 24 mammals. (A) The number of intact, truncated, and pseudo-genes is indicated in blue, yellow, and red, respectively. We treated the truncated genes as ‘putatively intact’. The dotted lines show the variation in the number of intact + ‘putatively intact’ genes among mammals. It should be noted that the number of *TAAR*s is obviously higher in megabats than in microbats. (B) Phylogenetic tree of intact *TAAR*s in 24 mammals. Only the intact genes were included in the tree. The *TAAR*s of the Egyptian fruit bat and Leschenault’s rousette are indicated by the square (green) and triangle (blue). It is obvious that the *TAAR*s of subfamilies seven and eight were expanded in two *Rousettus* bats. Zebrafish *TAAR*13c in the NCBI database was used as an outgroup. Mouse *TAAR*1-9 in the NCBI database was used as an indicator for each *TAAR* subfamily. Accession codes for these database-derived genes are available in Supplementary Fig. S4.

Our comparative genomic analyses of chemosensory receptor genes in the genomes of 25 mammals revealed that the copy number of the intact genes and pseudo-genes show a certain variation among bat species. In *T1R*s, the absence of *T1R1*, the umami receptor, in all of the bats that we analysed is consistent with the findings of the previous studies.^66^ All megabats possess two *T1R*s (*T1R2* and *T1R3*), whereas microbats are somewhat variable, in that they can possess no (greater false vampire bat), one (little brown bat), or two (Brandt’s bat, greater horseshoe bat) *T1R*s (Fig. 3A and Supplementary Table S5). It is noteworthy that all megabats possess *T1R2*, which is the sweet receptor, suggesting the importance of sweet taste for their frugivorous lifestyle. No intact *T1R*s in the greater false vampire bat could be explained by their specific adaptation for a carnivorous diet, which resembles the blood-feeding activity of the vampire bat (*Desmodus rotundus*), which also lost *T1Rs*.^66^^,^^69^

As for *T2R*s, which are bitter taste receptors, the copy numbers are relatively smaller in megabats than those in microbats (Fig. 3A and Supplementary Table S5). The smaller number of *T2R*s in megabats can also be explained by their frugivorous diet, as compared with that of microbats, which are mostly insectivores. Indeed, the repertoires of *T2R*s in primates have a strong correlation with their diet,^70^ suggesting the importance of *T2R*s for feeding adaptation in mammals.

We identified little variation between megabats and microbats in *FPR*s, which are expressed in the sensory neurons of the vomeronasal organ and mediate innate avoidance behaviours (Fig. 3ASupplementary Table S5).^71^ Suggesting that *FPR*-mediated chemo-detection is not directly linked with the difference in their habitats, mega- and microbats both possess two to eight *FPR*s. However, a previous study, by comparing the orthologous sequences among a broad range of mammals, found the signatures for the operation of positive selection in *FPR*s.^72^ Therefore, to examine the possible contribution of *FPR*s to the adaptive evolution of megabats, more detailed investigation is necessary by focussing on the *dN*/*dS* values among orthologous *FPR* sequences of many bat species, which are lacking at present.

There was an extensive reduction in *V1Rs*, which are known to be expressed in VNO neurons of mammals and detect various pheromones,^73–75^ in both megabats and microbats (Fig. 3A and Supplementary Table S5). Especially, only one *V1R* was found in the genomes of megabats. The reduction of *V1R*s revealed in this study is consistent with the findings of the previously published studies.^67^ The inactivation of *TRPC2*s^76^^,^^77^ and *ancV1R*s,^78^^,^^79^ which is responsible for VNO function, suggested the degeneration of VNOs in most bat lineages including megabats. Although most bats do not possess intact *V1R*s, Parnell’s mustached bat possesses four intact *V1R*s (Fig. 3A and Supplementary Table S5), which is consistent with the presence of the VNO in this species.^80^ In addition, recent study has suggested that there are a substantial number of *V1R*s in distantly related groups of phyllostomids and miniopterids, which possess an intact VNO, suggesting that they retained *V1R*-mediated chemical communication.^77^^,^^81^ Therefore, the ancestor of all extant bats is expected to possess an intact VNO, as well as a certain number of *V1R*s, that were independently degenerated after the divergence of each family, including megabats (Pteropodidae). Namely, the loss of echolocation and the degeneration of the VNO occurred spontaneously in the ancestor of megabats.

*V2R*s are expressed in the basal region of the VNO neurons^74^^,^^82^^,^^83^ and peptide pheromones were detected in mice.^84^^,^^85^ However, intact *V2R*s have been identified only in a limited number of mammals, such as rodents,^63^ mouse lemurs,^86^ and opossum.^87^ Our comprehensive analysis failed to find intact putative *V2R*s in the genomes of all bats and most of other mammals. This result suggests that, before the acquisition of the echolocation ability, the *V2R*-mediated pheromone detection system has already been lost in the common ancestor of all extant bat lineages. It is noteworthy that the hedgehog and the horse possess seven and one intact *V2R*s, respectively (Fig. 3A and Supplementary Table S5). This provides the first description of intact *V2R*s in the genomes of Laurasiatherian mammals. More detailed analyses may provide insight into the *V2R-*mediated pheromone detection system in these species.

One of the most intriguing results in the chemosensory receptor genes was obtained from *TAAR*s. Trace amine receptors have been believed to function as receptors for trace amines, for example, tyramine and octopamine in the brain.^88^ However, a recent study revealed that *TAAR*s may be expressed primarily or exclusively in the MOE,^89^ and are responsible for detecting volatile amines, including ethological odors that evoke innate animal behavioural responses.^90^ In this study, we revealed that the number of *TAAR*s was increased in the common ancestor of megabats. In particular, the number of *TAAR*s, which were identified to be from five to seven copies in microbats, increased to more than 15 copies in megabats. In particular, Leschenault’s rousette possess 38 putatively intact (29 intact and 9 truncated) *TAAR*s, which is the largest number identified among mammals (Fig. 3A;Supplementary Tables S5 and S6). The phylogenetic analyses of intact *TAAR*s for the 24 mammals clearly demonstrated that the expansion of the genes in the two *Rousettus* bats, including the Egyptian fruit bat and Leschenault’s rousette, occurred in subfamilies seven and eight in a species-specific manner (Fig. 3B;Supplementary Fig. S4 and Table S6). Eyun *et al*.^91^ also reported a high copy number of *TAAR*s in one megabat, the large flying fox; however, the repertoire was quite different from that of these two *Rousettus* bats (Fig. 3A;Supplementary Tables S5 and S6). Although *TAAR*s were expanded in subfamilies seven and eight in the two *Rousettus* species, they were expanded only in subfamily seven in the Java fruit bat. The number of intact genes, as well as the pseudo-genes, was highly variable among the megabats, suggesting that birth and death of *TAAR*s were quite active. Phylogenetic, as well as copy number, analyses suggest that *TAAR*s have provided a large contribution to some process of adaptive evolution and diversification of megabats. Interestingly, Pavlovich *et al*.^16^ revealed the gene expansion of *Mhc genes* in the genomes of the Egyptian fruit bat, suggesting novel modes of antiviral defense. Thus, the *Mhc* genes and *TAAR*s were both expanded in megabats. Santos *et al*.^92^ reported that *TAAR*s may be a key mediator in Mhc-dependent mating choices in the sac-winged bat (*Saccopteryx bilineata*). Based on these findings, it is possible that the megabats use diversified *TAAR*s for mate choice, by taking advantage of Mhc-related molecules that are also diversified. Functional experiments investigating *TAAR*s and mating in megabats may provide insight into the possible link between *TAAR*s and *Mhc* genes.

*OR*s, which are expressed in the MOE, have undergone extensive expansion and contraction that may be associated with environmental adaptations. In *OR*s, we also revealed the notable increase of the genes in megabats, which is more evident in two *Rousettus* bats, including the Egyptian fruit bat and Leschenault’s rousette (Fig. 3A and Supplementary Table S5). Although the copy numbers of putatively intact (intact and truncated) *OR*s span from 249 to 543 in microbats, those of megabats ranges from 401 to 740. The increase in the number of *OR*s in megabats may be the signature for the re-allocation in response, leading to the loss of the echolocation ability in the megabat ancestor. Hayden *et al*.^65^ identified convergent *OR* patterns linked to frugivorous diet in megabats and New World fruit-eating microbats (phyllostomids). Given that the increase in the *OR*s is more extensive, these patterns of *OR*s are not only linked to the frugivorous diet, but also to some other roles, such as predator avoidance and social communication.

By extensively analysing the copy-number variations of chemosensory receptor genes between megabats and microbats, we revealed obvious differences in *TAAR*s and *OR*s, both of which are expressed in the MOE. It is possible that the contraction of VNO-mediated chemo-detection and echolocation in megabats may lead to the expansion of chemo-detection genes expressed in the MOE. In addition, it is noteworthy that the repertoires of *TAAR*s and *OR*s were obviously differentiated even between closely related two species belonging to the *Rousettus*, suggesting that birth and death of these genes are quite active in this genus (Fig. 3A and B; Supplementary Tables S5 and S6). The results propose the possibility that two *Rousettus* bats are particularly dependent on olfaction through *TAAR*s and *OR*s.

### 3.5. Genes with elevated evolutionary rates in megabats

In addition to the candidate approach, which focussed on retroposons and chemosensory receptor genes, we also performed global analyses on the protein-coding genes of megabats. The elevation of *dN*/*dS* ratios were examined for the 6,365 single-copy orthologous genes^35^ using the branch model of codeml implemented in PAML4.8.^46^ The likelihood ratio tests and the inspection of *P*-value identified that the elevation of *dN*/*dS* ratios (*P *<* *0.05) was significant in 246 genes (Supplementary Table S7). As shown by the enrichment analyses for the resultant 246 genes using WebGESTALT,^93^ the elevation of the *dN*/*dS* ratios in megabats was remarkable in genes related to the immune system and protein catabolism (Table 1 and Supplementary Table S8).

**Table 1:** Gene list of immune response and protein catabolism with the elevation of *ω* of the *dN*/*dS* ratios in megabats

Symbol	Gene	***P*-value** ^a^	**Function** ^b^
*LYN*	LYN proto-oncogene, Src family tyrosine kinase	1.86E − 11	Immune system
*C8A*	Complement C8 alpha chain	3.62E − 06	Immune system
*PARP9*	Poly(ADP-ribose) polymerase family member 9	6.40E − 06	Immune system
*DHX36*	DEAH-box helicase 36	9.74E − 04	Immune system
*DHX9*	DEAH-box helicase 9	2.15E − 03	Immune system
*CD86*	CD86 molecule	6.82E − 03	Immune system, infection
*CD55*	CD55 molecule	1.05E − 02	Immune system, infection
*HK1*	Hexokinase 1	1.34E − 02	Immune system
*C8B*	Complement C8 beta chain	1.53E − 02	Immune system
*SEC14L1*	SEC14-like lipid binding 1	1.71E − 02	Immune system
*IL15*	Interleukin 15	2.44E − 02	Immune system, infection
*IL18*	Interleukin 18	3.00E − 02	Immune system, infection
*XBP1*	X-box binding protein 1	3.65E − 02	Immune system
*STK10*	Serine/threonine kinase 10	2.48E − 04	Immune system
*AP3B1*	Adaptor-related protein complex 3 subunit beta 1	1.53E − 02	Immune system
*CYLD*	CYLD lysine 63 deubiquitinase	2.08E − 02	Immune system
*IFNGR1*	Interferon gamma receptor 1	4.23E − 02	Immune system
*FAS*	Fas cell-surface death receptor	4.51E − 02	Immune system, infection
*CASP8*	Caspase 8	8.69E − 04	Infection
*HBS1L*	HBS1-like translational GTPase	2.01E − 02	Infection
*GNAL*	G protein subunit alpha L	1.49E − 02	Infection
*SNX9*	Sorting nexin 9	2.36E − 02	Infection
*AOX1*	Aldehyde oxidase 1	7.85E − 04	Protein catabolism
*TAT*	Tyrosine aminotransferase	9.47E − 03	Protein catabolism
*GSTZ1*	Glutathione-*S*-transferase zeta 1	3.24E − 02	Protein catabolism
*HADH*	Hydroxyacyl-CoA dehydrogenase	4.49E − 03	Protein catabolism
*CAT*	Catalase	5.55E − 03	Protein catabolism

a Statistical significance of likelihood ratio test for the elevation of *dN*/*dS* in megabat branches.

b Function was deduced by enrichment analysis in WebGESTALT.^93^

The elevation of the *dN*/*dS* ratios in immune system genes has been reported in several comparative genomic analyses on mammals, including the pangolin,^94^ microbat,^14^ and megabat.^16^ Notably, microbats and pangolins have recently begun to attract attention as possible host reservoirs of SARS-related coronaviruses responsible for the current outbreak of coronavirus disease-2019 (COVID-19).^95^^,^^96^ Pavlovich *et al*.^16^ revealed the episodic evolution of immune response genes in Egyptian rousette, a natural reservoir of Marburg virus, by showing an unusual expansion of NGK2, CD94, MHC, and IFN gene families. We revealed the episodic evolution by showing the elevation of *dN*/*dS* ratios in many immune response genes in megabat lineages (Table 1). The tolerance for zoonotic viruses without overt pathology in bats are consistent with the episodic evolution in immune response genes. Namely, co-evolution of viruses and immune system in these species may be facilitated by the adaptive evolution. Further molecular biological and physiological investigations of these candidate genes are of primary importance in elucidating how bats tolerate infections by various zoonotic viruses.

**Figure 4:**
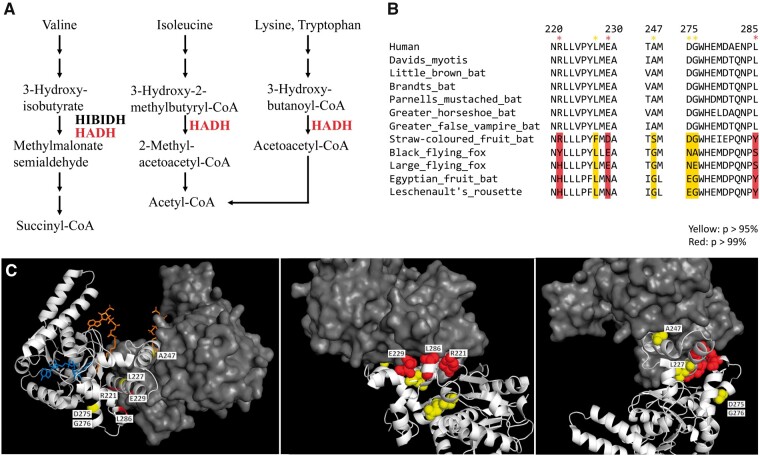
Positively selected sites in *HADH* on megabat lineages. (A) In protein metabolism, HADH is involved in the degradation of Ile, Val, Lys, Tyr and transforms these factors into acetyl-CoA or succinyl-CoA for the TCA cycle (https://www.genome.jp/dbget-bin/www_bget?hsa: 3033). (B) The sequence alignment between the positively selected sites in HADH in the megabat lineages and microbats and human HADH. The codon alignment of all HADH sequences used in this study is available in Supplementary Alignment File S3. The sites were identified by the branch-site model on PAML. Positively selected sites are highlighted in yellow (*P*, >95%) and red (*P*-value,p >99%). (C) Positively selected residues on megabat lineages are mapped on the human HADH dimer (PDB: 1F0Y). The A chain is presented as a spherical model (yellow and red). The HADH dimer A chain is shown as a cartoon model (white) and the B chain is shown as a surface model (gray). The ligands of HADH, NAD, and acetoacetyl-CoA are shown as a stick model (blue and orange, respectively).

Interestingly, the elevation of the *dN*/*dS* ratio of protein catabolism was also reported in the tyrosine aminotransferase gene (*TAT*) in megabats.^97^ To further investigate the evolution of the protein catabolism pathway in megabats, we focussed on another representative gene, 3-hydroxyacyl-CoA dehydrogenase (*HADH*), in which the elevation of the *dN*/*dS* ratio was significant in the branch model (Table 1; Supplementary Tables S9 and S10). *HADH* is involved in the degradation of Ile, Val, Lys, and Tyr to convert them into energy via the citric acid (TCA) cycle (Fig. 4A). The branch-site test for *HADH* (Fig. 4 and Supplementary Table S10) revealed that seven sites were positively selected with a posterior probability (*P*) of >95%, including three sites with a *P* of >99% (Fig. 4B). The likelihood for the operation of positive selection was not significant, as only a few sites were detected as positively selected (11%, Supplementary Table S10). We then mapped the positively selected sites on the human *HADH* dimer structure (PDB: 1F0Y, Fig. 4C). Although the positively selected sites were not located on the ligand (NAD and CAA) binding sites, it was of interest that four sites (R221, E229, A247, and L286) were located on the dimer interface (Fig. 4C). The mutations on these four residues change electric charges or polarities, such as R221Y, E229N, A247S, and L286S, suggesting that dimer formation is likely to be interrupted and enzyme catalysis is degraded. Shen *et al*.^97^ identified the significantly low activity of *TAT* in megabats and discussed that the elevation of the *dN*/*dS* ratio in *TAT* may be the relaxation of purifying selection in response to their frugivorous diet. Megabats may utilize the ingested proteins for the synthesis of new proteins, rather than for energy production through catabolism, as their diets, which include fruits and nectar, are rich in carbohydrates but poor in protein. Accordingly, it is possible that the megabats are less dependent on the protein catabolism pathway. In this study, we provide additional and inclusive evidence which suggests that the evolutionary constraints on genes for protein catabolism were relaxed due to the adaptation for frugivorous diets.

## 4. Conclusion

In summary, our comparative genomic analyses revealed several distinct signatures for adaptive evolution in megabats. (i) The activity of TEs is considerably lower compared to other mammals, which is possibly related to a defense mechanism against viruses. The small size of the genomes, which may be due to the low activity of TEs, could be advantageous in association with cellular metabolic constrains of flying organisms. (ii) *TAAR*s and *OR*s, which function in the neurons of MOE, show specific expansions, implying the important contribution of olfaction in their adaptation processes. (iii) Positive selection in genes for immunity may suggests the co-evolution of immune system and viruses, providing crucial insights into the mechanism of asymptomatic infection of bats for zoonotic viruses as a host reservoir. (iv) Positive selection in genes for protein catabolism is consistent with the ability of frugivorous feeding that is one of the adaptive characters specific to megabats.

## Supplementary data

The supplementary data are available at *DNARES* online.

## Supplementary Material

dsaa021_Supplementary_Data
