# Supplementary material for: Comparative genomic analyses illuminate the distinct evolution of megabats within Chiroptera
Source: DNA Res. 2020 Sep 23;27(4):dsaa021. doi: 10.1093/dnares/dsaa021 (PMC7547651; doi:10.1093/dnares/dsaa021)
Supplement: dsaa021_Supplementary_Data [file dsaa021_supplementary_data.zip › Figure S1, S2, S3, S4.pptx]

## Slide 1
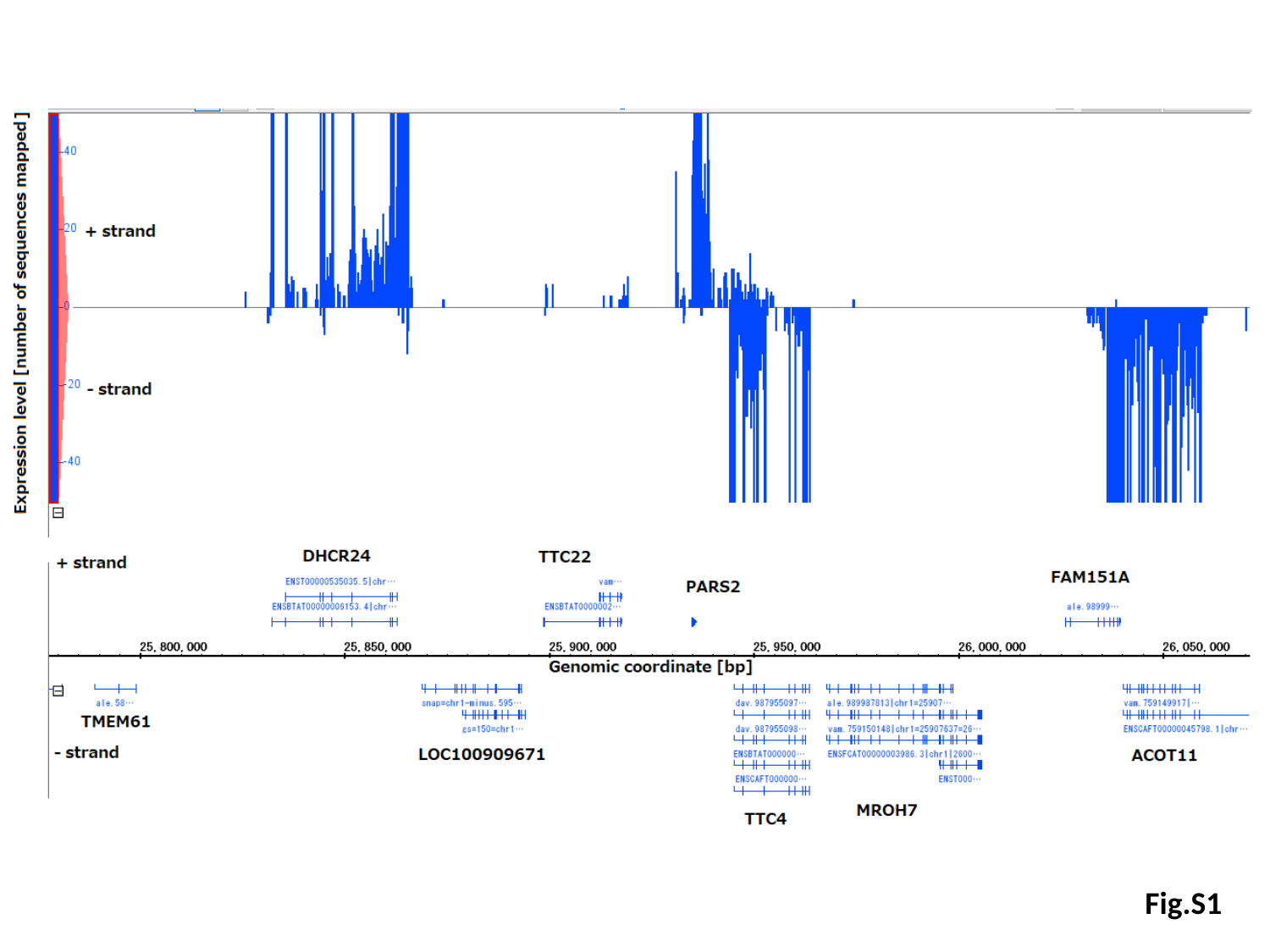

Fig.S1

## Slide 2
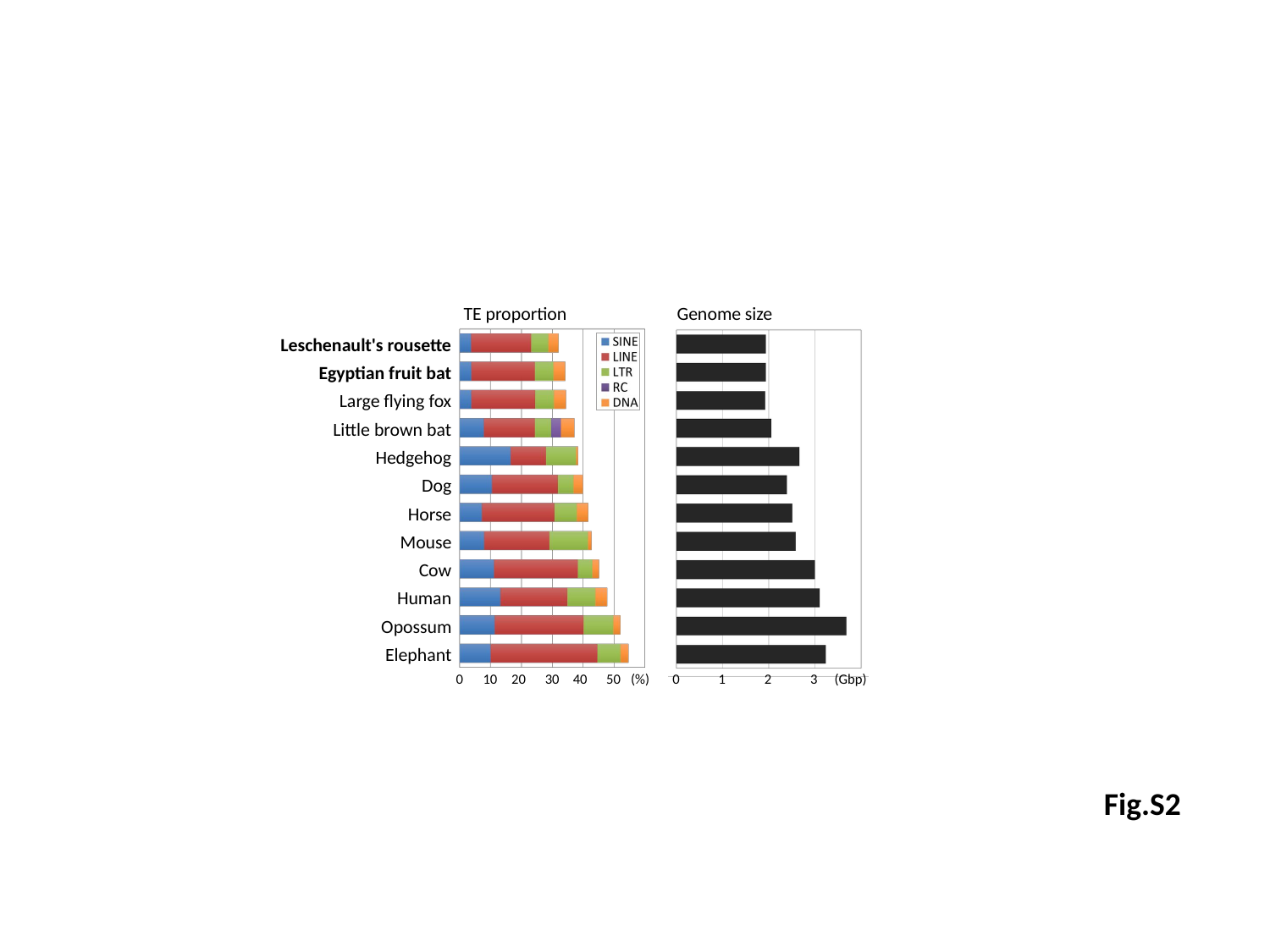

TE proportion
Genome size
Leschenault's rousette
Egyptian fruit bat
Large flying fox
Little brown bat
Hedgehog
Dog
Horse
Mouse
Cow
Human
Opossum
Elephant
0
10
20
30
40
50
(%)
0
1
2
3
(Gbp)
Fig.S2

## Slide 3
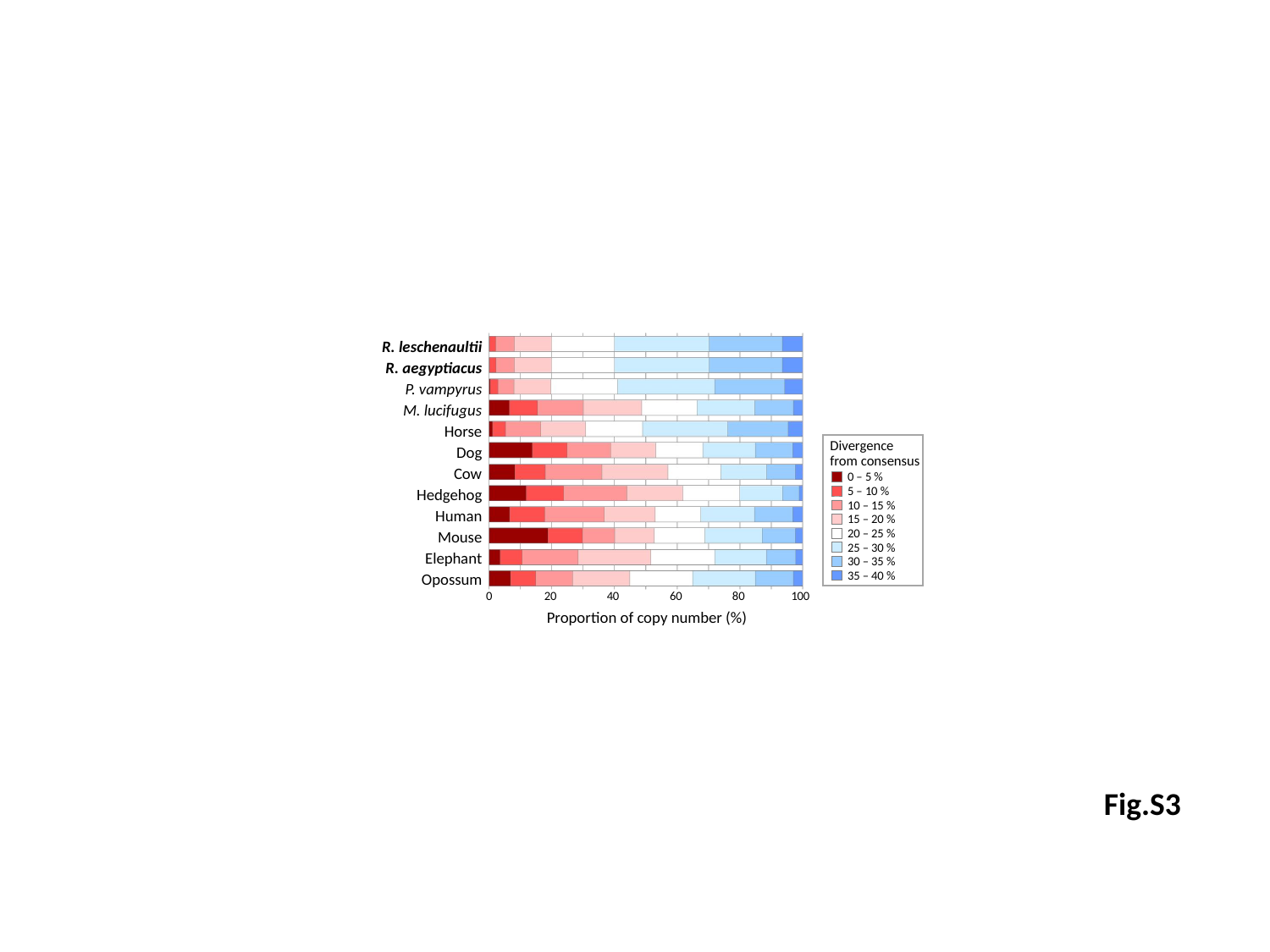

R. leschenaultii
R. aegyptiacus
P. vampyrus
M. lucifugus
Horse
Dog
Cow
Hedgehog
Human
Mouse
Elephant
Opossum
Divergence
from consensus
0 – 5 %
5 – 10 %
10 – 15 %
15 – 20 %
20 – 25 %
25 – 30 %
30 – 35 %
35 – 40 %
0
20
40
60
80
100
Proportion of copy number (%)
Fig.S3

## Slide 4
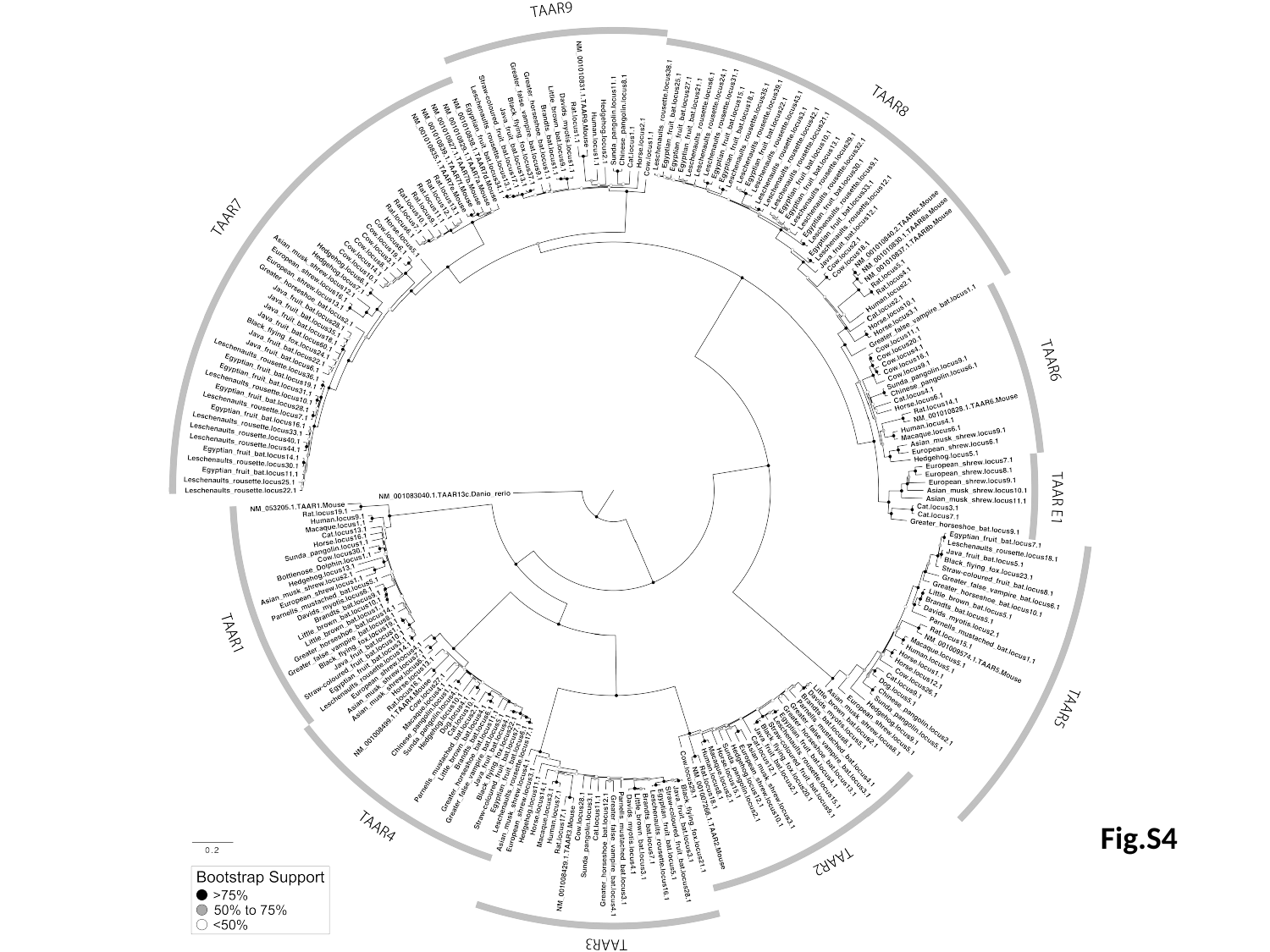

Fig.S4
