# Supplementary material for: Comparative genomic analyses illuminate the distinct evolution of megabats within Chiroptera
Source: DNA Res. 2020 Sep 23;27(4):dsaa021. doi: 10.1093/dnares/dsaa021 (PMC7547651; doi:10.1093/dnares/dsaa021)
Supplement: dsaa021_Supplementary_Data [file dsaa021_supplementary_data.zip › Table S1 to S10.pdf]

Table S1-1. The genome assembly statistics of Egyptian fruit bat and Leschenault's rousette

species	Assembler	Total size (excluding 'N')	number of Scaffolds	Scaffold N50	Contig N50	Longest
Egyptian fruit bat (*Rousettus aegyptiacus*)	Platanus v1.2.1	1.90 Gb	4,947	37.2 Mb	99.8 kb	145 Mb
Leschenault's rousette (*Rousettus leschenaultia*)	Platanus v1.2.1	1.90 Gb	8,141	32.7 Mb	35.2 Kb	96 M

* The statistics are calculated using scaffolds/contigs larger than 1kb.

Table S1-2. Information of the sequence library

Rousettus aegyptiacus

Library Type	Insert Size (bp)	Read length (bp)	Number of Reads	Number of Bases
Paired End	250	150	242 M	36.2 Gb
Paired End	450	150	741 M	111 Gb
Paired End	800	150	452 M	67.8 Gb
Mate Pair	3,000	150	305 M	30.5 Gb
Mate Pair	7,000	150	256 M	25.6 Gb
Mate Pair	10,000	150	295 M	44.3 Gb
Mate Pair	20,000	150	295 M	44.2 Gb

Rousettus leschenaultia

Library Type	Insert Size (bp)	Read length (bp)	Number of Reads	Number of Bases
Paired End	600	250	1523 M	381 G
Mate Pair	3000	150	231 M	34.7 G
Mate Pair	5000	150	218 M	32.7 G
Mate Pair	8000	150	229 M	34.3 G
Mate Pair	10000	150	228 M	34.3 G
Mate Pair	12000	150	229 M	34.4 G
Mate Pair	15000	150	230 M	34.5 G

Fosmid end Statistics

Number of fosmid clones	Strand	Number of H.Q. Reads	% of H.Q. Reads	Average Read Length	Average Clone Size
9,984	fwd	8,901	89.20%	785 bp	N.A.
	rev	8,888	89.00%	783 bp	N.A.
	both	8,750	87.60%	786 bp	36.7 kb

* H.Q. Reads are defined as reads which have >=100bp high quality region, excluding contamination.

Statistics of fosmid end mapping

Category	Number of cl	% of clones
Normal insert size (20kb - 5(	8,049	92.00%
Abnormal insert size	70	0.80%
Invalid strand on the same sc	34	0.40%
Different scaffolds	529	6.00%
Multi best hits	8	0.10%
Unmapped	60	0.70%

*Fosmid end sequences were mapped to the genome by using BLAT with parameters "-minScore=100 -maxIntron=1000 -noTrimA -fastMap".

Table S2. Species and classification of mammals used in the present comparative genome analyses

Superorder	Order		species	common name	genome assembly
Euarchontoglires	Primates		*Homo sapiens*	Human	GRCh38.p7
			Macaca mulatta	Macaque	Mmul_8.0.1
	Rodentia		*Mus musculus*	Mouse	GRCm38.p4
			Rattus norvegicus	Rat	Rnor_6.0
Laurasiatheria	Carnivora		*Felis catus*	Cat	Felis_catus_8.0
			Canis familiaris	Dog	CanFam3.1
	Pholidota		*M. pentadactyla*	Chinese pangolin	M_pentadactyla-1.1.1
			Manis javanica	Sunda pangolin	ManJav1.0
	Cetartiodactyla		*Tursiops truncatus*	Bottlenose Dolphin	NIST Tur_tru v1
			Bos taurus	Cow	Bos_taurus_UMD_3.1.1
	Perrisodactyla		*Equus caballus*	Horse	Equ Cab 2
	Eulipotyphla		*Erinaceus europaeus*	Hedgehog	EriEur2.0
			Suncus murinus	Asian musk shrew	r150910 (This study)
			Sorex araneus	Common shrew	SorAra2.0
	Chiroptera	Yangochiroptera	*Myotis davidii*	David's myotis	ASM32734v1
			Myotis lucifugus	Little brown bat	Myoluc2.0
			Myotis brandtii	Brandt's bat	ASM41265v1
			Pteronotus parnellii	Parnell's mustached bat	ASM46540v1
		Yinpterochiroptera	*Rhinolophus ferrumequinum*	Greater horseshoe bat	mRhiFer1_v1.p
			Megaderma lyra	Greater false vampire bat	ASM46534v1
			Eidolon helvum	Straw-coloured fruit bat	ASM46528v1
			Pteropus alecto	Black flying fox	ASM32557v1
			Pteropus vampyrus	Large flying fox	Pvam_2.0
			Rousettus aegyptiacus	Egyptian fruit bat	rouEgy_v1.4 (This study)
			Rousettus leschenaultii	Leschenault's rousette	rouLes150624 (This study)

Table S3. Genes determined in the six bat genomes

Species	Number of genes	Number of transcripts	Ratio of complete genes given BUSCO [%]
Rousettus_aegyptiacus	20,005	46,249	98
Rousettus_leschenaultii	20,913	47,073	97.8
Eidolon_helvum	20,272	31,441	76
Megaderma_lyra	19,296	28,367	53.8
Pteronotus_parnellii	19,654	29,653	63.8
Rhinolophus_ferrumequinum	20,229	31,222	71.1

Table S4. Contents of transposable elements in the *Rousettus* genomes

			Rousettus leschenaultii			*Rousettus aegyptiacus*		
Class	Clade/Superfamily	Group	Length (kbp)	Copies (x 10^3)	Coverage (%)	Length (kbp)	Copies (x 10^3)	Coverage (%)
SINE		MEG	2,844.3	17.0	0.15	2,857.8	16.9	0.15
		MEG-TR	600.8	3.2	0.03	597.6	3.2	0.03
		MEG-T2	570.9	2.5	0.03	572.2	2.5	0.03
		MIRs	69,411.2	459.9	3.62	69,267.4	458.7	3.65
		others	1,101.6	8.9	0.06	1,101.2	8.8	0.06
LINE	L1	Chiropteran L1	76,452.4	64.4	3.99	75,716.3	63.9	3.99
		Mammalian L1	184,105.4	297.4	9.60	183,845.3	296.3	9.68
		Chiropteran HAL1	28,299.0	21.1	1.48	28,302.7	21.1	1.49
		Mammalian HAL1	8,541.3	14.4	0.45	8,561.9	14.3	0.45
	L2	L2	85,887.2	310.3	4.48	85,810.4	309.6	4.52
	CR1	L3/CR1	9,801.6	44.3	0.51	9,797.4	44.3	0.52
	RTE	BovB/RTE	1,658.5	7.5	0.09	1,656.4	7.5	0.09
	others		3,059.8	10.7	0.16	3,065.8	10.7	0.16
LTR retrotransposons	ERV1	ERV1	28,160.3	60.8	1.47	27,966.2	60.4	1.47
	ERVL	ERVL	34,715.9	81.8	1.81	34,641.3	81.6	1.82
		MaLR	47,400.4	134.1	2.47	47,226.6	133.5	2.49
	ERVK	ERVK	1,656.9	2.7	0.09	1,681.8	3.0	0.09
	others/unknown		6,549.2	25.1	0.34	6,527.7	25.0	0.34
Rolling-circle transposons		Helitron	364.1	1.5	0.02	361.5	1.5	0.02
DNA transposons	TcMar	Tigger	18,247.9	71.9	0.95	18,211.4	71.8	0.96
		Mariner	884.5	5.6	0.05	885.3	5.6	0.05
		others	1,671.4	7.5	0.09	1,667.6	7.4	0.09
	hAT	Charlie	38,269.8	193.5	2.00	38,128.3	192.6	2.01
		Tip100	9,939.2	47.5	0.52	9,937.6	47.4	0.52
		others	5,837.0	33.4	0.30	5,839.1	33.4	0.31
	PiggyBac	PiggyBac	913.4	2.8	0.05	913.3	2.8	0.05
	others/unknown		1,451.5	9.8	0.08	1,341.6	9.1	0.07
unclassified			629.6	3.8	0.03	626.7	3.8	0.03
Total			669,025.0	1,943.5	34.88	667,108.4	1,936.6	35.13

Table S5. The copy number of chemo-receptor genes for 25 mammals (11 bats).

	T1R*			T2R			FPR			TAAR			V1R			V2R			OR		
	intact	truncate	pseudo	intact	truncate	pseudo	intact	truncate	pseudo	intact	truncate	pseudo	intact	truncate	pseudo	intact	truncate	pseudo	intact	truncate	pseudo
Human	3	0	0	53	0	30	4	0	1	5	0	3	4	0	139	0	0	18	398	0	442
Macaque	3	0	0	28	1	22	4	0	1	5	0	4	0	1	67	0	0	61	350	25	315
Mouse	3	0	2	36	0	16	8	0	3	15	0	3	222	2	148	131	0	349	1130	0	239
Rat	3	0	0	35	0	11	8	0	8	16	0	4	107	6	129	59	5	231	1369	39	834
Cat	2	0	1	12	0	14	2	0	1	10	0	4	27	0	87	0	0	14	808	85	298
Dog	3	0	0	15	0	12	2	0	1	2	0	2	9	0	46	0	0	12	816	4	276
Chinese pangolin	2	0	1	2	0	11	0	0	0	4	7	32	17	50	13	0	0	9	589	188	1038
Sunda pangolin	2	0	1	3	1	12	2	0	1	7	3	43	24	39	28	0	0	10	297	332	1403
Bottlenose Dolphin	0	0	3	0	0	10	2	0	1	0	0	2	2	1	31	0	0	7	12	1	156
Cow	3	0	0	18	1	12	0	0	0	19	0	12	39	0	42	0	0	16	1065	31	936
Horse	2	0	1	20	0	16	2	0	2	11	0	5	35	1	61	1	0	24	1060	18	1580
Hedgehog	3	0	0	21	0	18	2	0	1	9	0	3	61	4	45	7	2	23	977	65	176
Asian musk shrew	2	0	0	36	1	16	3	0	2	10	0	4	108	1	100	0	0	0	1209	19	225
Common shrew	2	0	0	42	4	7	1	1	1	11	0	6	52	7	46	0	0	0	1084	149	370
David's myotis	1	1	0	25	5	11	2	2	3	5	0	1	0	0	10	0	0	2	319	143	244
Little brown bat	1	0	0	28	2	9	4	0	3	7	0	3	0	0	13	0	0	1	530	13	162
Brandt's bat	2	1	0	25	6	6	3	5	2	6	0	3	0	0	18	0	0	2	453	90	151
Parnell's mustached bat	0	2	0	19	2	14	1	1	2	5	0	0	4	8	31	0	0	6	265	62	173
Greater horseshoe bat	2	0	1	17	0	7	2	0	3	8	0	6	0	0	27	0	0	8	389	0	194
Greater false vampire bat	0	0	2	12	5	11	1	1	4	7	0	3	1	2	37	0	0	4	199	50	179
Straw-coloured fruit bat	1	1	1	13	0	12	1	1	1	6	8	14	0	0	38	0	0	13	335	66	259
Black flying fox	2	0	1	14	0	8	1	4	3	7	9	24	0	0	37	0	0	15	392	129	366
Large flying fox	2	0	1	15	3	10	2	0	3	13	13	38	0	0	31	0	0	12	472	158	451
Egyptian fruit bat	2	0	1	12	1	15	2	1	4	22	3	9	1	0	37	0	0	12	611	55	364
Leschenault's rousette	2	0	1	16	1	11	2	0	5	29	9	10	1	0	38	0	0	10	680	60	376

※ T1Rs were judged by the sequence of transmembrane domain (exon 6). We treated the "truncate" squences as putatively "intact" in the graph (Fig. 3A).

Table S6. Number of TAAR genes in chiroptera

		assembly name	intact	truncate	pseudo	TAAR1	TAAR2	TAAR3	TAAR4	TAAR5	TAAR6	TAAR7	TAAR8	TAAR9	TAAR E1
Myotis davidii	David's myotis	ASM32734v1	5	0	1	1	1	1	0	1	0	0	0	1	0
Myotis lucifugus	Little brown bat	Myoluc2.0	7	0	3	2	1	1	1	1	0	0	0	1	0
Myotis brandtii	Brandt's bat	ASM41265v1	6	0	3	1	1	1	1	1	0	0	0	1	0
Pteronotus parnellii	Parnell's mustached bat	ASM46540v1	5	0	0	1	1	1	1	1	0	0	0	0	0
Rhinolophus ferrumequinum	Greater horseshoe bat	mRhiFer1_v1.p	8	0	6	1	1	1	1	1	0	1	0	1	1
Megaderma lyra	Greater false vampire bat	ASM46534v1	7	0	3	1	1	1	1	1	0	0	1	1	0
Eidolon helvum	Straw-coloured fruit bat	ASM46528v1	6	8	14	1	1	1	1	1	0	0	0	1	0
Pteropus alecto	Black flying fox	ASM32557v1	7	9	24	1	1	1	1	1	0	1(1)	0(7)	1	0(1)
Pteropus vampyrus	Large flying fox	Pvam_2.0	13	13	38	1	1	1	1	1(1)*	0	6(10)	1(2)	1	0
Rousettus aegyptiacus	Egyptian fruit bat	rouEgy_v1.4	22	3	9	1	1	1	1	1	0	6(2)	10(1)	1	0
Rousettus leschenaultii	Leschenault's rousette	rouLes150624	29	9	10	1	1	1	1	1	0	9(4)	14(5)	1	0

The number of intact genes were classified into subfamilies based on the phylogenetic analyses (Fig. 3B). *The number of truncated TAARs for each subfamily were indicated in parentheses.

Table S7. List of 246 genes with elevated dN/dS in megabats

gene Symbol	gene Name	entrezgene
DYNC1LI1	dynein cytoplasmic 1 light intermediate chain 1	51143
ECT2	epithelial cell transforming 2	1894
FUBP3	far upstream element binding protein 3	8939
STOML3	stomatin like 3	161003
LYN	LYN proto-oncogene, Src family tyrosine kinase	4067
TEX2	testis expressed 2	55852
UBAP2L	ubiquitin associated protein 2 like	9898
CNTLN	centlein	54875
ELAVL4	ELAV like RNA binding protein 4	1996
SYTL3	synaptotagmin like 3	94120
SLC38A11	solute carrier family 38 member 11	151258
CFAP47	cilia and flagella associated protein 47	286464
C8A	complement C8 alpha chain	731
MAN1A1	mannosidase alpha class 1A member 1	4121
ACCS	1-aminocyclopropane-1-carboxylate synthase homolog (inactive)	84680
PARP9	poly(ADP-ribose) polymerase family member 9	83666
QRSL1	QRSL1, glutaminyl-tRNA amidotransferase subunit A	55278
KRT23	keratin 23	25984
ERICH3	glutamate rich 3	127254
SH2D4B	SH2 domain containing 4B	387694
KITLG	KIT ligand	4254
METTL8	methyltransferase like 8	79828
ADAM2	ADAM metallopeptidase domain 2	2515
PPIP5K1	diphosphoinositol pentakisphosphate kinase 1	9677
HMGN3	high mobility group nucleosomal binding domain 3	9324
ETV6	ETS variant 6	2120
ABHD12B	abhydrolase domain containing 12B	145447
ARHGAP4	Rho GTPase activating protein 4	393
SEC14L2	SEC14 like lipid binding 2	23541
FAM135B	family with sequence similarity 135 member B	51059
TMEM86A	transmembrane protein 86A	144110
PRKCE	protein kinase C epsilon	5581
PLEKHS1	pleckstrin homology domain containing S1	79949
STK10	serine/threonine kinase 10	6793
GPN3	GPN-loop GTPase 3	51184
CCDC83	coiled-coil domain containing 83	220047
PIGA	phosphatidylinositol glycan anchor biosynthesis class A	5277
SPHKAP	SPHK1 interactor, AKAP domain containing	80309
ZZEF1	zinc finger ZZ-type and EF-hand domain containing 1	23140
EIF2AK1	eukaryotic translation initiation factor 2 alpha kinase 1	27102
DMGDH	dimethylglycine dehydrogenase	29958

HERC1	HECT and RLD domain containing E3 ubiquitin protein ligase family member 1	8925
ANKRD1	ankyrin repeat domain 1	27063
MRPL49	mitochondrial ribosomal protein L49	740
SLC25A24	solute carrier family 25 member 24	29957
AOX1	aldehyde oxidase 1	316
PADI4	peptidyl arginine deiminase 4	23569
OTUD6B	OTU domain containing 6B	51633
CASP8	caspase 8	841
KIF24	kinesin family member 24	347240
ZNF704	zinc finger protein 704	619279
DHX36	DEAH-box helicase 36	170506
ALG5	ALG5, dolichyl-phosphate beta-glucosyltransferase	29880
LRGUK	leucine rich repeats and guanylate kinase domain containing	136332
PPM1E	protein phosphatase, Mg2+/Mn2+ dependent 1E	22843
LRRIQ1	leucine rich repeats and IQ motif containing 1	84125
RNF149	ring finger protein 149	284996
CDC5L	cell division cycle 5 like	988
BRD8	bromodomain containing 8	10902
SYAP1	synapse associated protein 1	94056
DNAH8	dynein axonemal heavy chain 8	1769
KIAA1755	KIAA1755	85449
LSM1	LSM1 homolog, mRNA degradation associated	27257
LRRC36	leucine rich repeat containing 36	55282
DHX9	DExH-box helicase 9	1660
SYTL2	synaptotagmin like 2	54843
PMCH	pro-melanin concentrating hormone	5367
MAP3K15	mitogen-activated protein kinase kinase kinase 15	389840
USP2	ubiquitin specific peptidase 2	9099
SI	sucrase-isomaltase	6476
BTC	betacellulin	685
SAMD4A	sterile alpha motif domain containing 4A	23034
DTNB	dystrobrevin beta	1838
COQ6	coenzyme Q6, monooxygenase	51004
AFAP1L2	actin filament associated protein 1 like 2	84632
PARM1	prostate androgen-regulated mucin-like protein 1	25849
HCRTR2	hypocretin receptor 2	3062
NR1I3	nuclear receptor subfamily 1 group I member 3	9970
VEZT	vezatin, adherens junctions transmembrane protein	55591
CREG1	cellular repressor of E1A stimulated genes 1	8804
EVA1C	eva-1 homolog C	59271
GIPC2	GIPC PDZ domain containing family member 2	54810
QSER1	glutamine and serine rich 1	79832
BRCA1	BRCA1, DNA repair associated	672
HADH	hydroxyacyl-CoA dehydrogenase	3033
PHACTR2	phosphatase and actin regulator 2	9749

CCDC30	coiled-coil domain containing 30	728621
MMAB	metabolism of cobalamin associated B	326625
MYO6	myosin VI	4646
FAN1	FANCD2 and FANCI associated nuclease 1	22909
DSP	desmoplakin	1832
ITGB5	integrin subunit beta 5	3693
KDELC2	KDEL motif containing 2	143888
MNS1	meiosis specific nuclear structural 1	55329
CAT	catalase	847
VLDLR	very low density lipoprotein receptor	7436
TBC1D31	TBC1 domain family member 31	93594
SLC12A1	solute carrier family 12 member 1	6557
CD86	CD86 molecule	942
MYOM3	myomesin 3	127294
ALPK2	alpha kinase 2	115701
EREG	epiregulin	2069
GLIPR1L2	GLIPR1 like 2	144321
MTFR2	mitochondrial fission regulator 2	113115
LPIN1	lipin 1	23175
AKAP13	A-kinase anchoring protein 13	11214
BRDT	bromodomain testis associated	676
CERS3	ceramide synthase 3	204219
TNC	tenascin C	3371
DIAPH1	diaphanous related formin 1	1729
FKTN	fukutin	2218
GRPR	gastrin releasing peptide receptor	2925
HMBS	hydroxymethylbilane synthase	3145
TOM1L1	target of myb1 like 1 membrane trafficking protein	10040
NHS	NHS actin remodeling regulator	4810
PRR11	proline rich 11	55771
ANTXR2	ANTXR cell adhesion molecule 2	118429
PPARGC1B	PPARG coactivator 1 beta	133522
CLCA2	chloride channel accessory 2	9635
EGFLAM	EGF like, fibronectin type III and laminin G domains	133584
EPHX2	epoxide hydrolase 2	2053
TBC1D4	TBC1 domain family member 4	9882
TAT	tyrosine aminotransferase	6898
MAP4	microtubule associated protein 4	4134
ZDHHC13	zinc finger DHHC-type containing 13	54503
CCDC178	coiled-coil domain containing 178	374864
GSN	gelsolin	2934
CD55	CD55 molecule (Cromer blood group)	1604
WDTC1	WD and tetratricopeptide repeats 1	23038
VRK3	VRK serine/threonine kinase 3	51231
WBP4	WW domain binding protein 4	11193

ZBBX	zinc finger B-box domain containing	79740
DUSP27	dual specificity phosphatase 27, atypical	92235
SLC25A48	solute carrier family 25 member 48	153328
CEP68	centrosomal protein 68	23177
ENAH	ENAH, actin regulator	55740
MAB21L3	mab-21 like 3	126868
PRKG2	protein kinase cGMP-dependent 2	5593
MEFV	MEFV, pyrin innate immunity regulator	4210
HK1	hexokinase 1	3098
NGEF	neuronal guanine nucleotide exchange factor	25791
SFMBT1	Scm like with four mbt domains 1	51460
QRICH2	glutamine rich 2	84074
MFSD1	major facilitator superfamily domain containing 1	64747
MGA	MGA, MAX dimerization protein	23269
GPR107	G protein-coupled receptor 107	57720
RREB1	ras responsive element binding protein 1	6239
GNAL	G protein subunit alpha L	2774
CLIP1	CAP-Gly domain containing linker protein 1	6249
E2F6	E2F transcription factor 6	1876
AP3B1	adaptor related protein complex 3 subunit beta 1	8546
C8B	complement C8 beta chain	732
SLC16A1	solute carrier family 16 member 1	6566
RBM28	RNA binding motif protein 28	55131
RND2	Rho family GTPase 2	8153
HPCA	hippocalcin	3208
CYP24A1	cytochrome P450 family 24 subfamily A member 1	1591
FYCO1	FYVE and coiled-coil domain containing 1	79443
NOXRED1	NADP dependent oxidoreductase domain containing 1	122945
RRN3	RRN3 homolog, RNA polymerase I transcription factor	54700
DDRGK1	DDRGK domain containing 1	65992
TARBP1	TAR (HIV-1) RNA binding protein 1	6894
LEXM	lymphocyte expansion molecule	163747
TTK	TTK protein kinase	7272
SEC14L1	SEC14 like lipid binding 1	6397
TMEM45B	transmembrane protein 45B	120224
HHIP	hedgehog interacting protein	64399
KIAA1107	KIAA1107	23285
CYFIP2	cytoplasmic FMR1 interacting protein 2	26999
XRRA1	X-ray radiation resistance associated 1	143570
TTC33	tetratricopeptide repeat domain 33	23548
KIAA0100	KIAA0100	9703
HBS1L	HBS1 like translational GTPase	10767
SPERT	spermatid associated	220082
BUB1	BUB1 mitotic checkpoint serine/threonine kinase	699
CYLD	CYLD lysine 63 deubiquitinase	1540

DIAPH3	diaphanous related formin 3	81624
DDX55	DEAD-box helicase 55	57696
TMIGD1	transmembrane and immunoglobulin domain containing 1	388364
SH3TC2	SH3 domain and tetratricopeptide repeats 2	79628
CRB1	crumbs cell polarity complex component 1	23418
SNX9	sorting nexin 9	51429
MRC2	mannose receptor C type 2	9902
FAM193A	family with sequence similarity 193 member A	8603
CDCA2	cell division cycle associated 2	157313
TAX1BP1	Tax1 binding protein 1	8887
IL15	interleukin 15	3600
GPATCH1	G-patch domain containing 1	55094
ANKRD27	ankyrin repeat domain 27	84079
CEP83	centrosomal protein 83	51134
SFR1	SWI5 dependent homologous recombination repair protein 1	119392
SERINC5	serine incorporator 5	256987
UBR2	ubiquitin protein ligase E3 component n-recognin 2	23304
GLP2R	glucagon like peptide 2 receptor	9340
CDK17	cyclin dependent kinase 17	5128
ARID3C	AT-rich interaction domain 3C	138715
BRINP1	BMP/retinoic acid inducible neural specific 1	1620
SERPINC1	serpin family C member 1	462
STEAP4	STEAP4 metalloreductase	79689
IL18	interleukin 18	3606
ST5	suppression of tumorigenicity 5	6764
ITPKC	inositol-trisphosphate 3-kinase C	80271
LRIG1	leucine rich repeats and immunoglobulin like domains 1	26018
STAG3	stromal antigen 3	10734
GSTZ1	glutathione S-transferase zeta 1	2954
SETDB1	SET domain bifurcated histone lysine methyltransferase 1	9869
PRSS54	serine protease 54	221191
NDRG1	N-myc downstream regulated 1	10397
BTBD1	BTB domain containing 1	53339
KIAA0232	KIAA0232	9778
AHCTF1	AT-hook containing transcription factor 1	25909
TMEM252	transmembrane protein 252	169693
MSRA	methionine sulfoxide reductase A	4482
EXPH5	exophilin 5	23086
DSG3	desmoglein 3	1830
XBP1	X-box binding protein 1	7494
SRRM2	serine/arginine repetitive matrix 2	23524
TUBD1	tubulin delta 1	51174
CSPP1	centrosome and spindle pole associated protein 1	79848
ST6GALNAC6	ST6 N-acetylgalactosaminide alpha-2,6-sialyltransferase 6	30815
ALDH1A2	aldehyde dehydrogenase 1 family member A2	8854

FBXO15	F-box protein 15	201456
KBTBD8	kelch repeat and BTB domain containing 8	84541
IFNGR1	interferon gamma receptor 1	3459
ESPL1	extra spindle pole bodies like 1, separase	9700
NOL10	nucleolar protein 10	79954
MOV10L1	Mov10 like RISC complex RNA helicase 1	54456
SLC8A3	solute carrier family 8 member A3	6547
RANGRF	RAN guanine nucleotide release factor	29098
FUCA1	alpha-L-fucosidase 1	2517
SLC16A4	solute carrier family 16 member 4	9122
CENPI	centromere protein I	2491
CCDC175	coiled-coil domain containing 175	729665
FAS	Fas cell surface death receptor	355
POLH	DNA polymerase eta	5429
WDR63	WD repeat domain 63	126820
VGLL1	vestigial like family member 1	51442
LYPD6B	LY6/PLAUR domain containing 6B	130576
GCG	glucagon	2641
RHAG	Rh associated glycoprotein	6005
TYRO3	TYRO3 protein tyrosine kinase	7301
FAM124A	family with sequence similarity 124 member A	220108
TDRD9	tudor domain containing 9	122402
TM2D3	TM2 domain containing 3	80213
CNBD2	cyclic nucleotide binding domain containing 2	140894
TMX4	thioredoxin related transmembrane protein 4	56255

Table S8. The result of enrichment analyses for genes with elevated dN/dS on the branch of megabat ancestor

Description	overlapped genes (symbol)
**GO (Biological_Process, Cellular_Component, Molecular_Function)**	
regulation of immune effector process	LYN;C8A;PARP9;DHX36;DHX9;CD86;CD55;HK1;C8B;SEC14L1;IL15;IL18;XBP1
meiotic cell cycle	MNS1;EREG;BRDT;TTK;BUB1;UBR2;STAG3;ESPL1;MOV10L1;TDRD9
organelle fission	DYNC1LI1;BTC;EREG;MTFR2;BRDT;TOM1L1;TTK;BUB1;UBR2;STAG3;ESPL1;MOV10L1;TDRD9
intracellular receptor signaling pathway	CASP8;BRD8;NR1I3;BRCA1;PPARGC1B;CYP24A1;DDRGK1;SEC14L1;CYLD;ALDH1A2
regulation of cell division	ECT2;PRKCE;BTC;CAT;EREG;AHCTF1;CSPP1
peptidyl-tyrosine modification	LYN;PARP9;KITLG;PRKCE;BTC;AFAP1L2;EREG;TTK;IL15;IL18;TYRO3
positive regulation of cell adhesion	LYN;PRKCE;CD86;EGFLAM;CD55;RREB1;AP3B1;CYLD;IL15;IL18;XBP1
microtubule	DYNC1LI1;ARHGAP4;KIF24;DNAH8;MAP4;MEFV;CLIP1;CYLD;NDRG1;TUBD1;CSPP1;SLC8A3
regulation of peptide secretion	LYN;HMGN3;PRKCE;ANKRD1;DHX36;DHX9;HADH;HK1;SLC16A1;EXPH5;XBP1;GCG
leukocyte cell-cell adhesion	LYN;STK10;CD86;CD55;AP3B1;CYLD;IL15;IL18;XBP1
**KEGG pathway**	
Ubiquinone and other terpenoid-quinone biosynthesis	COQ6;TAT
Tyrosine metabolism	AOX1;TAT;GSTZ1
Platinum drug resistance	CASP8;BRCA1;FAS;POLH
Tryptophan metabolism	AOX1;HADH;CAT
Complement and coagulation cascades	C8A;CD55;C8B;SERPINC1
Fanconi anemia pathway	BRCA1;FAN1;POLH
Legionellosis	CASP8;HBS1L;IL18
Viral myocarditis	CASP8;CD86;CD55
Chagas disease (American trypanosomiasis)	CASP8;GNAL;IFNGR1;FAS
Long-term depression	LYN;PRKG2
**Disease (OMIM, Disgenet, GLAD4U)**	
Leishmaniasis, Visceral	SNX9;IL15;IL18;IFNGR1
Autosomal recessive predisposition	C8A;DMGDH;CASP8;SI;COQ6;HADH;MYO6;FAN1;DSP;CAT;VLDLR;SLC12A1;LPIN1;DIAPH1;FKTN;TAT;MEFV;HK1;AP3B1;C8B;SLC16A1;RBM28;HPCA;CYP24A1;CRB1;CEP83;SERPINC1;STAG3;NDRG1;EXPH5;IFNGR1;FUCA1;POLH;RHAG
Meningitis	C8A;MEFV;C8B;FAS
Myalgia	LPIN1;HMBS;MEFV;RREB1;FAS
Skin Diseases, Genetic	CASP8;BRCA1;DSP;CAT;HMBS;PPARGC1B;MEFV;AP3B1;CYLD;ITPKC;DSG3;POLH
Oral Ulcer	CAT;MEFV;FAS
Hemolysis	C8A;PIGA;CD55;HK1;C8B
Tyrosinemias	TAT;GSTZ1
Sunburn	CAT;MSRA;POLH
Constipation	BRCA1;FAN1;SLC12A1;HMBS;MEFV;RREB1;GCG
**Phenotype**	
Pulmonary embolism	PIGA;CD55;MEFV;SERPINC1;FAS
Abnormal auditory evoked potentials	DIAPH3;SH3TC2;NDRG1
Diarrhea	CASP8;SI;BRCA1;HADH;SLC12A1;HMBS;ANTXR2;CD55;MEFV;IFNGR1
Anorexia	BRCA1;HMBS;MEFV;IFNGR1;FAS
Increased IgG level	IFNGR1;FAS
Oral ulcer	CAT;MEFV;FAS
Recurrent aspiration pneumonia	SLC25A24;CSPP1
Vitiligo	KITLG;FAS
Peritonitis	BRCA1;MEFV
Myalgia	LPIN1;FKTN;HMBS;MEFV;RREB1;FAS

The categories for immune system, infection, and protein catabolism were highlighted by gray, orange and yellow, respectively

Table S9. Akaike Information Criterion (AIC) comparisons of different codon frequency models in codeml for HADH.

	np[a]	lnL	AIC[b]	⊿AIC	Relative weight[c]
CF0	0	-7845.3	15690.5	0	1
CF1	3	-7857.0	15720.1	-29.6	3.8E-07
CF2	9	-7866.1	15750.3	-59.8	1.1E-13
CF3	60	-7881.5	15883.1	-192.5	1.6E-42

Abbreviations,
CF0 = frequencies for each codon are assumed to be equal; CF1 = codon frequencies are calculated from average nucleotide frequencies; CF2 = codon frequencies are calculated from average nucleotide frequencies at each of three codon positions; CF3 = codon frequencies are treated as free parameters.

[a] Number of free parameters

[b] AIC = 2*np – 2*lnL

[c] Relative weight = exp(0.5*ΔAIC)

Table S10. Summary of the results of selection analyses and likelihood ratio tests for *HADH*

Branch model

		np	lnL	branch category	dN/dS	N*dN	S*dS
HADH	model0	0	-7845.3	background	0.17		
	model1	1	-7829.2	background	0.15	630.9	1669.4
				stem and crown Pteropodidae	0.41	84.6	81.6
	model2	2	-7828.0	background	0.15	631	1668.5
				stem Pteropodidae	0.27	20.9	30.2
				crown Pteropodidae	0.48	63.8	52

	p-value
model0 vs model1	1.4E-08
model1 vs model2	0.12

Branch-site model

	lnL		purifying	neutral	positive
null	-7704.9	proportion	0.69	0.31	not allowed
		dN/dS	0.082	1	not allowed
alternative	-7703.1	proportion	0.75	0.14	0.11
		dN/dS	0.084	1	2.09

	p-value
null vs alt.	0.056

	Positively selected site
p > 95%	L227, A247, D275, G276
p > 99%	R221, E229, L286
